# CHCHD4 (MIA40) and the mitochondrial disulfide relay system

**DOI:** 10.1042/BST20190232

**Published:** 2021-02-18

**Authors:** Hasan Al-Habib, Margaret Ashcroft

**Affiliations:** Department of Medicine, University of Cambridge, Cambridge Biomedical Campus, Hills Road, Cambridge CB2 0QQ, U.K.

**Keywords:** cancer, CHCHD4, oxidoreductase, disulfide relay system, hypoxia, metabolism, mitochondria, mitochondrial import

## Abstract

Mitochondria are pivotal for normal cellular physiology, as they perform a crucial role in diverse cellular functions and processes, including respiration and the regulation of bioenergetic and biosynthetic pathways, as well as regulating cellular signalling and transcriptional networks. In this way, mitochondria are central to the cell's homeostatic machinery, and as such mitochondrial dysfunction underlies the pathology of a diverse range of diseases including mitochondrial disease and cancer. Mitochondrial import pathways and targeting mechanisms provide the means to transport into mitochondria the hundreds of nuclear-encoded mitochondrial proteins that are critical for the organelle's many functions. One such import pathway is the highly evolutionarily conserved disulfide relay system (DRS) within the mitochondrial intermembrane space (IMS), whereby proteins undergo a form of oxidation-dependent protein import. A central component of the DRS is the oxidoreductase coiled-coil-helix-coiled-coil-helix (CHCH) domain-containing protein 4 (CHCHD4, also known as MIA40), the human homologue of yeast Mia40. Here, we summarise the recent advances made to our understanding of the role of CHCHD4 and the DRS in physiology and disease, with a specific focus on the emerging importance of CHCHD4 in regulating the cellular response to low oxygen (hypoxia) and metabolism in cancer.

## Introduction

Mitochondria are organelles whose essential functions include the generation of adenosine triphosphate (ATP) through respiration and the regulation of bioenergetic and biosynthetic pathways [1]. They not only perform a key role in cellular processes (e.g. metabolism, apoptosis and autophagy), but are also involved in regulating important cell signalling functions, including the response to cellular stressors such as low oxygen (hypoxia) [1,2].

**Figure 1.**
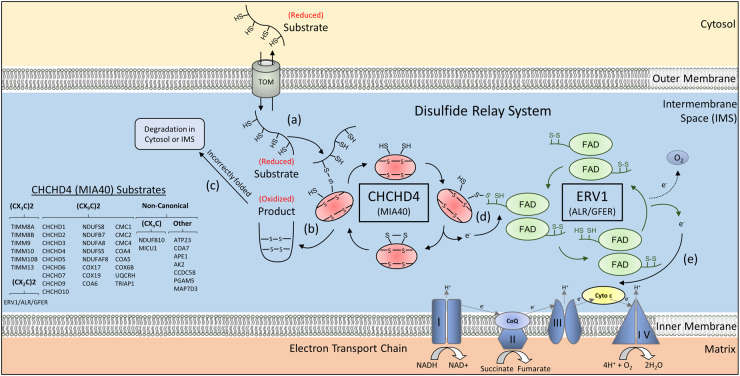
CHCHD4 (MIA40) and the disulfide relay system. (**a**) Nuclear-encoded DRS substrates carrying (CX_3_C)2, (CX_9_C)2 or other cysteine configurations (see table) are translated in the cytosol and imported into the IMS through the TOM complex in a reduced state. Substrates interact with CHCHD4 (also known as MIA40, shown in red) as they translocate via the TOM. CHCHD4 forms a transient intermolecular disulfide bond with the substrate protein. (**b**) CHCHD4 catalyses the formation of intramolecular disulfide bonds within the substrate, ultimately resulting in oxidation of the substrate, which can then become properly folded. Multiple rounds of oxidation and isomerisation of disulfide bonds may be required for certain substrates. Once correctly oxidised and folded, the product proteins can carry out their proper functions within the IMS, and are no longer capable of re-localising back into the cytosol. (**c**) Substrates that are incorrectly folded become degraded, either in the cytosol by the proteosome or in the IMS by proteases. (**d**) Electrons generated by oxidation of the substrate are transferred to the CPC motif of CHCHD4, which is now in a reduced state. ERV1 (also known as ALR or GFER, shown in green) regenerates oxidised CHCHD4 by accepting the electrons gained by the CPC motif of CHCHD4. (**e**) These electrons are shuffled within ERV1 via the FAD domain, from where the electrons become preferentially transferred to cytochrome c (Cyto c) and then to complex IV (IV) of the electron transport chain. Alternatively, electrons are transferred to molecular oxygen. Table (left) shows a range of known CHCHD4 (MIA40) substrates, identified in yeast and humans. Abbreviations: ALR, augmenter of liver regeneration; CHCHD4, coiled-coil-helix-coiled-coil-helix (CHCH) domain-containing protein 4; CoQ, coenzyme Q; Cyto c, cytochrome complex; ERV1, essential for respiration and viability 1; FAD, flavin adenine dinucleotide; GFER, growth factor, augmenter of liver regeneration (ERV1 homolog, *S. cerevisiae*); MIA40, mitochondrial intermembrane space import and assembly protein 40; NAD+, nicotinamide adenine dinucleotide; NAD(H): nicotinamide adenine dinucleotide (hydrogen); TOM, translocase of the outer membrane.

Mitochondria consist of an outer and inner mitochondrial membrane (OMM and IMM) that encompass the intermembrane space (IMS) and surround a central matrix. While mitochondria contain a genome encoding 13 well-characterised proteins that are essential for mitochondrial function, there are also hundreds of nuclear-encoded proteins that are imported into mitochondria following their translation in the cytosol [3]. Mitochondrial import pathways and targeting mechanisms have evolved to transport different proteins from the cytosol to the appropriate mitochondrial location [3]. One such pathway is the disulfide relay system (DRS) within the IMS [4–7] (Figure 1).

The central component of the DRS, first discovered in yeast (*Saccharomyces cerevisiae*), is Mia40 (Tim40) [5,8], an oxidoreductase that imports small cysteine-containing substrates, typically containing a twin CX_3_C or CX_9_C motif, into the IMS [7]. Mia40 recognises an IMS-targeting signal (ITS, also known as MISS) — a nine amino acid sequence found within its substrates [9,10] that assists the docking of one substrate cysteine with Mia40's redox-active cysteine–proline–cysteine (CPC) motif. Once covalently bound to the CPC [11,12], Mia40 introduces intramolecular disulfide bonds into its substrates, thus folding and stabilising them within the IMS. Critical to Mia40 function is another DRS component, the FAD-dependent sulfhydryl oxidase Erv1 (ERV1/ALR/GFER in humans), which acts to re-oxidise Mia40 following a reaction between the oxidoreductase and a substrate, thereby ensuring it is capable of further enzymatic reactions [6,7,13,14]. This re-oxidation of Mia40 involves the transfer of electrons to Erv1, which are passed to cytochrome c and then to complex IV (CIV) of the electron transport chain (ETC) [15] (Figure 1).

The human homologue of Mia40, which is highly evolutionarily conserved, was cloned [4] and described as coiled-coil-helix-coiled-coil-helix (CHCH) domain-containing protein 4 (CHCHD4, also known as MIA40) [16]. *CHCHD4* in humans has been shown to code for two differentially spliced isoforms [4,16], designated as variant 1 (*CHCHD4.1*) and variant 2 (*CHCHD4.2*). *CHCHD4.1* was found to be ubiquitously expressed in human tissues and across a panel of human cell lines, whereas *CHCHD4.2* was shown to be differentially expressed [16]. CHCHD4 (MIA40) is CHCHD4.1, and in comparison, CHCHD4.2 contains a longer N-terminus and does not contain a cysteine at position four (C4). CHCHD4.2 was shown to localise to mitochondria [16], however further work is required to understand its specific function.

The importance of CHCHD4 and the DRS is evidenced by its conserved essentiality in yeast, mice and zebrafish [5,17,18], as well as the essentiality of its substrate proteins, which regulate diverse mitochondrial and cellular functions, including mitochondrial (mt)DNA and proteome maintenance, oxidative phosphorylation (OXPHOS), and intracellular oxygenation. Indeed, recent studies indicate that CHCHD4 acts on a greater range of substrates and performs a wider variety of functions than previously thought [19,20], leading to the DRS being described as the ‘sorting hub’ of the IMS [21]. Moreover, a study has shown that, in yeast, Mia40 could not be substituted by corresponding components from other cellular compartments, for example the endoplasmic reticulum (i.e. Pdi1) [22]. This study demonstrates that Mia40 is a unique, indispensable and essential component of mitochondrial biogenesis, and concludes that DRS development marks a critical step in eukaryotic evolution [22].

Studies have shown that dysregulation of the DRS underlies mitochondrial disease [23–27] and cancer [16,20,28–30]. Here, we summarise the recent advances made to our understanding of the role of CHCHD4 and the DRS in physiology and disease, with a specific focus on the emerging importance of CHCHD4 in regulating hypoxia signalling and metabolism in cancer.

## The DRS is evolutionarily conserved

The oxidoreductase Mia40, first discovered in yeast [5,8,31], later in humans [4] and with homologues in plants, animals and fungi [7] represents the central component of the DRS, catalysing the oxidative folding of small (typically <25 kDa) nuclear-encoded mitochondrial proteins. Much of our understanding of the molecular workings of the DRS has come from yeast studies [5,6,8,13,14,31]. Substrate proteins enter the IMS through the translocase of the outer membrane (TOM) protein complex of the OMM and simultaneously interact with the oxidised form of Mia40 [7,32–34], which has been shown to occur via a transient intermolecular disulfide bond [7]. This leads to the oxidation of the substrate, eventually enabling the proper folding of the protein required to perform its function, as well as trapping it within the IMS and preventing re-localization to the cytosol [7] (Figure 1). Notably, for certain substrates, oxidation can proposedly occur via an intramolecular disulfide isomerisation mechanism [35]. An alternative to this model comes from observations suggesting that hydrophobic interactions between Mia40 and the substrate ITS/MISS, rather than the formation of an intermolecular disulfide bond between the two proteins, trap the substrate within the IMS, a model supported by the finding that the redox-active CPC motif appears to be required for substrate folding but not import [7]. Once the introduction of intramolecular disulfide bonds and substrate oxidative folding has been mediated by Mia40, Erv1 can transfer its gained electrons to various electron acceptors. The primary acceptor is cytochrome c, linking the DRS to the ETC [13,15] (Figure 1). An *in vitro* study has shown that the mammalian homologue of Erv1 (ALR), can transfer electrons to oxygen itself, resulting in measurable hydrogen peroxide release [36]. In yeast, the soluble fumarate reductase Osm1 acts as an electron acceptor in anaerobic conditions [37].

Cellular compartments outside the IMS also play a role in facilitating the oxidative folding of substrates in the DRS. For example, in the cytosol of human cells, glutaredoxins utilise the glutathione pool to ensure that CHCHD4 substrates are maintained in a reduced state, thus enabling import into the IMS. The same function is performed by the thioredoxin system in yeast cytosol [7,38–42]. Within the IMS, the glutathione pool and glutaredoxins perform reduction of misfolded mixed-disulfide intermediates between CHCHD4 and substrates. To prevent this proofreading mechanism from inhibiting the correct folding of substrates by CHCHD4, glutaredoxin levels in the IMS are limited. This enables a form of kinetic restriction, whereby trapped intermediates are reduced and prevented from blocking the TOM complex pore, but at a slower rate in the IMS compared with the cytosol, therefore still allowing efficient oxidation of DRS substrates [40,43].

Glutaredoxins are also involved in iron sulfur (Fe-S) cluster mitochondrial trafficking [44,45]. CHCHD4 has been shown to play a vital role in Fe-S cluster export from the mitochondria, for which the CPC motif is critical [46,47]. Four cysteine residues within CHCHD4 were demonstrated to undergo reversible glutathionylation, three of which are located within the twin CX_9_C motif [47]. Thiriveedi et al. [47] described a model whereby CHCHD4, through a combination of its oligomerisation, Fe-S cluster binding, and reversible S-glutathionylation capacities, can act as a repository for mitochondrial reactive oxygen species (ROS) and transfer electrons directly to cytochrome c. The authors suggest that CHCHD4 may facilitate the flow of electrons through the ETC by quenching ROS generated at complex III (CIII) then directing them to CIV via cytochrome c [47]. ROS homeostasis is crucial because aberrant mitochondrial production of ROS is associated with cellular damage and a variety of pathologies [48].

From yeast to higher eukaryotes, CHCHD4 homologues share a highly evolutionarily conserved CHCH domain, containing four conserved cysteine residues within a twin CX_9_C motif, connected to the redox-active CPC motif which contains two conserved cysteine residues. The structures of both human and yeast CHCHD4/Mia40 proteins have been solved [11,49–51].

The core CHCH domain consists of a helix-loop-helix structure in a coiled-coil configuration, stabilised by a pair of disulfide bridges. Together the helices shape a hydrophobic pocket in the centre of the protein, forming a docking site for substrates. Attached is a flexible helical arm containing the CPC motif. The cysteine residues of the CPC motif can form intermolecular disulfide bridges with the substrate protein before the CPC motif becomes reduced itself, catalysing the formation of a disulfide bridge within the substrate [21]. The cysteines of the CHCH domain and CPC motif are required for localisation of the protein to the IMS in both yeast [4,11,52] and humans [16,53]. Furthermore, the C-terminus of CHCHD4 is highly negatively charged, an evolutionarily conserved feature, which has been shown in human cells to be important for protection from proteasomal degradation in the cytosol and its post-translational import into the IMS [54].

As mentioned above, Erv1 functions to regenerate oxidised Mia40 by accepting the gained electrons from the reduced CPC motif, and contains a twin CX_2_C motif [6,13,14,21,55–59]. Erv1 is a homodimer, with each subunit consisting of 2 domains: an unstructured N-terminal domain that mimics a substrate upon binding to Mia40 [56] and the FAD-binding domain, from which the electrons are transferred (Figure 1). Despite being mostly homologous in structure, Erv1 monomers dimerise through non-covalent interactions in yeast, whereas disulfide bridges exist between the equivalent monomers in ALR [60].

While the working components of the DRS were first described in yeast [5,6,8,13,14,31], they have been subsequently shown to be functionally conserved in higher organisms [4,11,17,18,27,51–53,59]. This functional conservation is underscored by the fact that the key components of the DRS are essential across species: loss of *Chchd4* has been shown to be lethal in mice [17] and zebrafish [18], as it was shown in yeast [5,8]. Similarly, loss of *Erv1*/*Alr* is lethal in both mice [30] and yeast [14].

## CHCHD4 in humans

Human CHCHD4 contains key differences compared with yeast Mia40. Although the central part of the protein, containing the catalytically and structurally important cysteines, is well conserved across eukaryotic homologues, the homology outside this area is relatively low (∼20%) [4,11]. Human CHCHD4 is smaller (∼21 kDa) than yeast Mia40 (∼40 kDa) [4], which contains an N-terminal transmembrane region that functions as a sorting signal for insertion of Mia40 into the IMM via a stop-transfer mechanism [61]. Human CHCHD4 lacks this N-terminal membrane anchor, and is therefore soluble in the IMS [4,11,17,18,51–53].

CHCHD4 import into the IMS has been shown to be dependent on apoptosis-inducing factor (AIF), a flavoprotein that also has a key role in cell survival, proliferation and differentiation [27,62]. AIF has been shown to bind to the N-terminal 27 residues of CHCHD4 [17], requiring its cofactor NADH to do so [63,64], and AIF depletion results in reduced CHCHD4 protein levels [17,27]. However, the precise manner of their interaction or how AIF facilitates CHCHD4 import into the IMS remains unclear [17,27]. Interestingly, loss of AIF has been shown to result in loss of ETC complex I (CI) levels and activity [65–68], although it seems that its role in CI biogenesis is limited to its facilitation of CHCHD4 import, since overexpression of CHCHD4 or import of CHCHD4 independent of AIF has been shown to render AIF dispensable for CI biogenesis [17,69]. A recent study in Drosophila suggests that compromised import of the Mia40 substrate MIC19 (a component of the mitochondrial contact site and cristae organising system) in the context of loss of Drosophila AIF (dAIF) is an underlying mechanism for dAIF affecting CI biogenesis [70].

## CHCHD4 (MIA40) substrates and mitochondrial function

Previously, CHCHD4 (MIA40) substrates have been described as containing an evolutionarily conserved twin CX_3_C or CX_9_C motif structurally configured within two antiparallel alpha-helices [43]. After undergoing oxidation by CHCHD4, these helices are joined by disulfide bridges (Figure 1). Notably, ERV1/ALR/GFER contains a twin CX_2_C motif, and other substrates contain alternative CX_n_C configurations (Figure 1, see table). Recent studies have expanded the diversity of CHCHD4 substrates beyond those containing the canonical cysteine configuration, both in yeast [71,72] and mammalian [19,73–75] systems (Figure 1, see table). In yeast, the protease Atp23 is oxidised across multiple iterations of Mia40 binding, and therefore contains multiple Mia40 binding sites [71]. In humans, the ETC assembly factor COA7 has neither an ITS nor cysteines arranged in a CX_n_C motif [76], whilst the calcium uniporter protein MICU1 is imported via an alternate pathway, but oxidation by CHCHD4 results in an intermolecular disulfide bridge between MICU1 and its inhibitory paralog MICU2 [19,77]. Such discoveries have indicated CHCHD4 substrates undergo oxidation for other purposes beyond import and folding [21]. Furthermore, variation in the dependence of DRS substrates on CHCHD4 expression levels for import into the IMS has also been observed [20].

CHCHD4 (MIA40) substrates are involved in a range of mitochondrial functions. These include subunits and/or assembly factors of CI, CIII and CIV of the ETC [19,20]. Moreover, CHCHD4 substrates are required for efficient transport of proteins across the IMM, namely subunits of the translocase of the inner mitochondrial membrane (e.g. TIMM9, TIMM10). Additionally, a number of CHCHD4 substrates function in the regulation of mitochondrial ultrastructure, including the formation of mitochondrial cristae and the organisation of the IMM [70,78]. Interestingly, a study in zebrafish demonstrated that homozygous loss of *chchd4a*, the essential homologue of *CHCHD4*, resulted in enlarged mitochondrial structures, and reduced basal oxygen consumption rate (OCR) in *chchd4a* targeted zebrafish embryos [18]. Furthermore, investigations into the underlying mechanisms of various mitochondrial disorders have revealed new CHCHD4 (MIA40) substrates, including the CI subunits NDUFB10 [23,41,65] and NDUFA8 [79], CHCHD10 [78] and COA7 [76,80,81].

Collectively, these studies demonstrate how crucial both the components and substrates of the DRS pathway are to the biogenesis of various mitochondrial proteins and complexes, and the correct function of the ETC, which are important for many physiological processes.

## CHCHD4, hypoxia signalling and cancer

**Figure 2.**
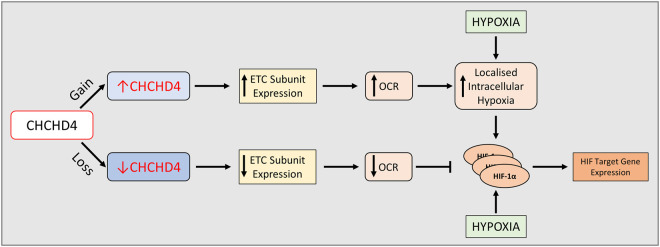
CHCHD4 regulates intracellular oxygenation. Gain of CHCHD4 (↑CHCHD4), refers to overexpression of CHCHD4 (using CHCHD4.1), which leads to an increase in ETC subunit expression, OXPHOS and basal cellular OCR. This results in localised intracellular hypoxia in tumour cells and leads to HIF-1α stabilisation and activation which can be enhanced further in hypoxia. Conversely, loss of CHCHD4 (↓CHCHD4), refers to knockdown of CHCHD4 (using shRNA targeting both *CHCHD4* variants), which leads to a decrease in ETC subunit expression and basal cellular OCR. This results in a reduction in HIF-1α stabilisation and HIF activation in hypoxia. Abbreviations: CHCHD4, coiled-coil-helix-coiled-coil-helix domain-containing protein 4; ETC, electron transport chain; HIF-1α, hypoxia-inducible factor-1α; HIF, hypoxia-inducible factor; OCR, oxygen consumption rate OXPHOS, oxidative phosphorylation.

In cancer, mitochondria contribute to adaptive metabolic responses that enable tumour cells to survive and metastasise [82]. Through its central role in controlling ETC function via the DRS, CHCHD4 has been shown to regulate basal cellular OCR, intracellular oxygenation [83] and hypoxia signalling in tumour cells [16,83] (Figure 2). Thus, CHCHD4 is primely positioned to influence tumour cell biology.

**Figure 3.**
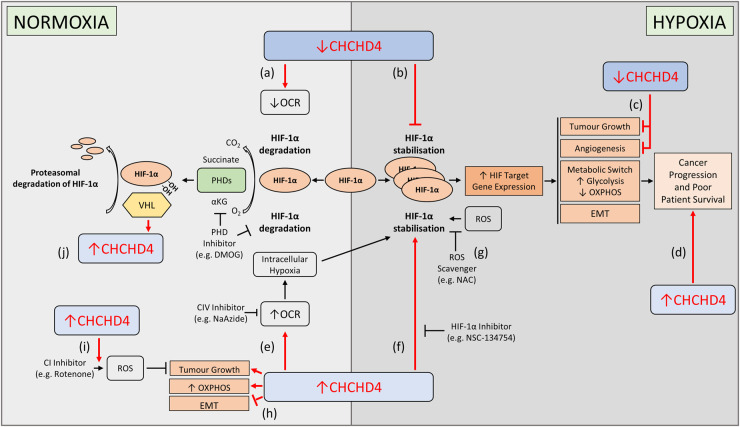
CHCHD4 and hypoxia (HIF) signalling in cancer. CHCHD4 regulates hypoxia (HIF) signalling in cancer through different routes as illustrated. ↑CHCHD4, refers to overexpression of CHCHD4 (using CHCHD4.1), and ↓CHCHD4, refers to knockdown of CHCHD4 (using shRNA targeting both *CHCHD4* variants). (**a**,**b**) CHCHD4 is required for basal cellular OCR and HIF-1α stabilisation. CHCHD4 knockdown significantly reduces basal cellular OCR in normoxia (**a**), and blocks HIF-1α stabilisation and target gene expression in tumour cells in response to hypoxia (**b**). HIF-1α stabilisation in hypoxia was not reduced by shRNA knockdown of CHCHD4 in the presence of the PHD inhibitor DMOG. (**c**) CHCHD4 is required for tumour growth and angiogenesis. CHCHD4 knockdown blocks tumour growth and angiogenesis *in vivo*. (**d**) High *CHCHD4* expression in breast, colon and glioma patient tumours significantly correlates with the hypoxia gene signature, proliferative pathways including mTORC1, increased cancer progression and poor patient survival. (**e–g**) Overexpression of CHCHD4 in tumour cells increases basal cellular OCR and intracellular hypoxia (**e**), and results in enhanced HIF-1α stabilisation in hypoxia (**f**), all of which are blocked by the CIV inhibitor sodium azide (NaAzide). Hypoxia-induced reactive oxygen species (ROS) production contributes to HIF-1α stabilisation, which is abrogated by ROS scavengers. CHCHD4-mediated enhanced HIF-1α stabilisation in hypoxia is not affected by ROS scavengers (e.g. *N*-acetyl cysteine) (**g**), but is blocked by the small molecule HIF-α inhibitor NSC-134754. (**h**) Overexpression of CHCHD4 in tumour cells significantly increases tumour growth in normoxia and hypoxia, promoting increased OXPHOS in normoxia yet enhancing glycolysis in hypoxia, as well as negatively regulating EMT-related phenotypes in normoxia. (**i**) Overexpression of CHCHD4 renders tumour cells more sensitive to growth inhibition by CI inhibitors, at least in part by increased ROS production. (**j**) VHL regulates CHCHD4 expression in renal carcinoma cells (RCC). CHCHD4 expression in RCC is up-regulated by reconstitution of VHL through a mechanism separable from VHL's regulation of HIF-1α. Abbreviations: αKG, α-ketoglutarate; CHCHD4, coiled-coil-Helix-coiled-coil-Helix domain containing 4; DMOG, dimethyloxalylglycine; ETC, electron transport chain; EMT, epithelial-mesenchymal transition; HIF-1α, hypoxia-inducible factor-1α; OCR, oxygen consumption rate; NAC, *N*-acetylcysteine; PHDs, prolyl hydroxylase domain enzymes; ROS, reactive oxygen species; VHL, von Hippel-Lindau tumour suppressor.

Hypoxia-inducible factor (HIF) transcription factors orchestrate the cellular response to hypoxia [2]. Under normoxic conditions, HIF-1α is hydroxylated by prolyl hydroxylase domain (PHD) enzymes, allowing recruitment of the von Hippel-Lindau (VHL) E3 ubiquitin ligase complex and targeting of HIF-1α for proteasomal degradation. In hypoxia, hydroxylation is inhibited, relieving HIF-1α degradation by VHL and leading to its stabilisation. HIF-1α translocates to the nucleus and binds to HIF-1β, forming the HIF complex. HIF transactivates >150 diverse target genes, including those involved in regulating tumour growth, angiogenesis, metabolism, and epithelial to mesenchymal transition (EMT) phenotypes in cancer (Figure 3). Hypoxia and HIF-1α overexpression is observed across human cancers, correlating with disease progression and poor patient survival [84].

Interestingly, high *CHCHD4* expression in breast cancer and glioma patient tumours was shown to significantly correlate with the hypoxia gene signature, metastasis and decreased survival of patients with these cancers [16,83], suggesting a critical role for CHCHD4 in regulating hypoxia signalling and cancer progression. Consistently, shRNA knockdown of CHCHD4 was shown to significantly block HIF-1α stabilisation and HIF target gene expression in tumour cells in response to hypoxia, and block tumour growth and angiogenesis *in vivo* [16,83] (Figures 2, 3). Conversely, overexpression of CHCHD4 in tumour cells was shown to increase basal cellular OCR and intracellular hypoxia [83], and led to enhanced HIF-1α stabilisation in hypoxia [16], all of which were blocked by the CIV inhibitor sodium azide [16,83] (Figures 2, 3). Furthermore, overexpression of CHCHD4 was shown to provide a proliferative advantage to tumour cells in both normoxia and hypoxia, proposedly due to CHCHD4 expression stimulating the most efficient utilisation of glucose depending on oxygen levels [16,20].

Increased expression of *CHCHD4* across a range of cancer types has been shown to correlate with OXPHOS and proliferative pathways including mTORC1 [28,85,86]. Consistently, overexpression of CHCHD4 in tumour cells was shown to increase both CI-activity and mTORC1 activation, as well as affecting glutamine metabolism [28]. Indeed, CHCHD4 overexpressing cells were shown to exhibit increased sensitivity to the growth-inhibiting effects of CI inhibitors [20], which could be rescued by either supplementation of cells with exogenous aspartate, or expression of a rotenone-insensitive yeast NADH-dehydrogenase (Ndi1) [28]. Therefore, CHCHD4 appears to regulate tumour cell proliferation, in part through its effects on CI-mediated metabolism, via a proposed mechanism whereby CHCHD4 influences amino acid stimulation of mTORC1-dependent protein synthesis, thus promoting tumour cell proliferation [28].

Mitochondrial ROS production has been shown to contribute to HIF-1α stabilisation in hypoxia [87–90], which is abrogated by ROS scavengers, such as *N*-acetylcysteine [91]. Interestingly, CHCHD4-mediated enhanced HIF-1α stabilisation in hypoxia was not affected by *N*-acetylcysteine [16], suggesting ROS is not involved. ROS-independent modulation of mitochondrial OCR at CIV, as a response to hypoxia, has been suggested to result in redistribution of intracellular oxygen, leading to PHD inactivation and HIF-1α stabilisation [92]. In line with this, HIF-1α stabilisation by the PHD inhibitor DMOG was not affected by shRNA knockdown of CHCHD4 in hypoxia [16], while CHCHD4-mediated HIF-1α stabilisation in hypoxia was blocked by the small molecule HIF-α inhibitor NSC-134754 [20] (Figure 2). However, mitochondrial ROS is involved in how CHCHD4 regulates tumour cell survival and growth, specifically in the context of CI inhibitors [20]. Loss of CHCHD4 was shown to protect tumour cells from the growth inhibitory effects of CI inhibitors, whilst overexpression of CHCHD4 was shown to confer significant sensitivity to CI inhibitors, partly through increased mitochondrial ROS production [21] (Figure 3). Given the involvement of ROS in redox signalling [48], further insight into the role of ROS in CHCHD4 function will be of particular interest.

In cancer, EMT phenotypes are associated with metastasis [93]. CHCHD4 has been shown to negatively regulate the expression of genes related to EMT phenotypes [28], which seems contrary to the finding that increased *CHCHD4* expression in tumours is associated with tumour progression in patients with certain cancer types [16]. One explanation may be that, in tumours with up-regulated CHCHD4, increased proliferation leads to outgrowing of the vascular supply of oxygen, promoting hypoxia, a known driver of EMT phenotypes [94]. HIF signalling is then initiated and activates the transcription of EMT genes, ultimately leading to metastatic dissemination of tumour cells. Indeed, shRNA knockdown of CHCHD4 has been shown to reduce tumour cell invasion in hypoxia [16].

Further work is required to elucidate the precise molecular mechanism(s) for how CHCHD4 controls HIF activation, hypoxia signalling and cancer progression in specific cancer types. In fact, a recent study examined the effects of VHL reconstitution on CHCHD4 function in renal carcinoma cells (RCC), since RCC exhibit loss of VHL function, and consequently HIF is constitutively activated [29]. Interestingly, CHCHD4 expression, ETC subunit expression, basal OCR, and cellular ATP in RCC were up-regulated by reconstitution of VHL through a mechanism separable from its regulation of HIF-1α [29] (Figure 3).

Notably, other components of the DRS have also been shown to be involved in certain cancers. For example, ERV1/ALR/GFER has been linked to hepatocellular carcinoma (HCC), with one study showing that a liver-specific knockout in mice caused accelerated development of HCC [30]. Furthermore, AIF-mediated regulation of mitochondrial respiration and OXPHOS has been shown to drive tumour progression in a mouse model of lung cancer [95].

The emerging role of CHCHD4 and the DRS in hypoxia signalling and cancer progression could provide new avenues for therapeutic intervention.

## Perspectives

- Importance of the field: CHCHD4 and the DRS regulate the import of many cysteine-containing proteins into the IMS that are required for normal physiological function. Dysregulation of the DRS, either through mutation of CHCHD4 (MIA40) substrates or changes in *CHCHD4* expression, underlies pathophysiology associated with mitochondrial disease and cancer, respectively.
- Current thinking: The essentiality of CHCHD4 and ERV1/ALR/GFER is well established, as is their crucial role within the DRS. Recent studies have shed light on the emerging range of CHCHD4 (MIA40) substrates, as well as the differences between yeast and human DRS systems. Additionally, the role of CHCHD4 in cellular signalling pathways, such as the hypoxia pathway, illustrates the diverse scope of its functions.
- Future directions: Identifying the differences in dependency on CHCHD4, its substrates and the DRS between tissues will likely be the subject of future studies. Also, understanding the significance of having two different *CHCHD4* variants in humans, as well as further insight into the molecular mechanisms underlying the apparently separable extramitochondrial functions of CHCHD4, will be an investigative goal going forward.

## Open Access

Open access for this article was enabled by the participation of University of Cambridge in an all-inclusive *Read & Publish* pilot with Portland Press and the Biochemical Society under a transformative agreement with JISC.

## Abbreviations

AIF: apoptosis-inducing factor
ATP: adenosine triphosphate
CHCH: coiled-coil-helix-coiled-coil-helix
CHCHD4: coiled-coil-helix-coiled-coil-helix (CHCH) domain-containing protein 4
CI: complex I
complex III: CIII
complex IV: CV
CPC: cysteine–proline–cysteine
DRS: disulfide relay system
EMT: epithelial to mesenchymal transition
Erv1: essential for respiration and viability 1
ETC: electron transport chain
FAD: flavin adenine dinucleotide
GFER: growth factor, augmenter of liver regeneration (ERV1 homolog, *S. cerevisiae*)
HCC: hepatocellular carcinoma
HIF: hypoxia-inducible factor
IMM: inner mitochondrial membrane
IMS: intermembrane space
ITS: IMS-targeting signal
Mia40: mitochondrial intermembrane space import and assembly protein 40
MISS: mitochondrial intermembrane space sorting signal
mTORC1: mammalian target of rapamycin complex 1
OCR: oxygen consumption rate
OMM: outer mitochondrial membrane
OXPHOS: oxidative phosphorylation
PHD: prolyl hydroxylase domain
RCC: renal carcinoma cells
ROS: reactive oxygen species
TOM: translocase of the outer membrane
VHL: von Hippel-Lindau
